# Successful treatment of *Rhizopus arrhizus* rhino-orbital-cerebral mucormycosis with isavuconazole salvage therapy following extensive debridement

**DOI:** 10.1016/j.mmcr.2021.03.005

**Published:** 2021-03-17

**Authors:** R.P. Miller, L. Farrugia, J. Leask, K. Khalsa, N. Khanna, L. Melia

**Affiliations:** a Department of Ear, Nose & Throat (ENT) Surgery, Queen Elizabeth University Hospital, Glasgow, UK; b Department of Microbiology, Queen Elizabeth University Hospital, Glasgow, UK; c Depatment of Urology, Queen Elizabeth University Hospital, Glasgow, UK

**Keywords:** Rhino-orbital-cerebral mucormycosis, Diabetes mellitus, Isavuconazole, *Rhizopus arrhizus*

## Abstract

A 61-year old lady with poorly-controlled type 2 diabetes mellitus was diagnosed with rhino-orbital-cerebral mucormycosis following presentation with sinusitis, ophthalmoplegia, proptosis and facial numbness. She was treated successfully with aggressive surgical intervention including orbital exenteration, accompanied by anti-fungal therapy with liposomal amphotericin B and posaconazole, followed by isavuconazole as salvage therapy. We discuss the challenges around optimising antifungal therapy of this lethal infection in the context of hepatic and renal toxicity.

## 1 Introduction

Mucormycosis is a life-threatening fungal infection characterised by acute onset and manifested by a range of clinical syndromes, of which rhino-orbital-cerebral (ROCM) and pulmonary infections are the most common [1].The causative fungi are classified in the order *Mucorales*; of which the genera *Rhizopus, Mucor* and *Rhizomucor* are most commonly implicated; with genera *Lichtheimia* (formerly *Absidia*)*, Saksenaea, Apophysomyces* and *Cunninghamella* also encountered [2]. Of these, the most common causative agent of ROCM is *Rhizopus arrhizus* [3].

Many mucoraceous moulds are saprophytic aerobic fungi, acquired environmentally via inhalation of spores. Spores then commonly colonise the nose, oral mucosa, throat or paranasal sinuses, and may act as opportunistic pathogens in those with predisposing factors, most commonly diabetes mellitus, neutropenia or concurrent malignancy. Hyperglycaemia within the context of diabetes mellitus facilitates germination, hyphae formation and subsequent angioinvasion and local tissue spread [4].

We report a case of ROCM in a 61-year-old with poorly-controlled type 2 diabetes mellitus (T2DM), with a focus on the challenges around optimising antifungal therapy in a patient experiencing significant drug toxicity.

## 2 Case presentation

A 61-year old white British lady was referred to the local ENT department (day 0) with an acute history of photophobia, diplopia and right-sided facial numbness, preceded by rhinorrhoea and right maxillary sinus pain and swelling. Her past medical history included chronic obstructive pulmonary disease, with recent treatment for an infective exacerbation with a short course of prednisolone and doxycycline, as well as poorly-controlled T2DM (HbA1c: 134mmol/mol). The patient had no previous history of sinusitis.

On examination, she was noted to have a right-sided facial droop, facial swelling, numbness over the maxillary division of the trigeminal nerve and weakness of muscles innervated by the marginal mandibular nerve. Reduced visual acuity and ophthalmoplegia of the right eye were also noted. On initial flexible nasoendoscopy, pus and crusting of the right nasal cavity with oedema of the maxillary meatus, but no tissue necrosis, were observed.

**Fig. 1:**
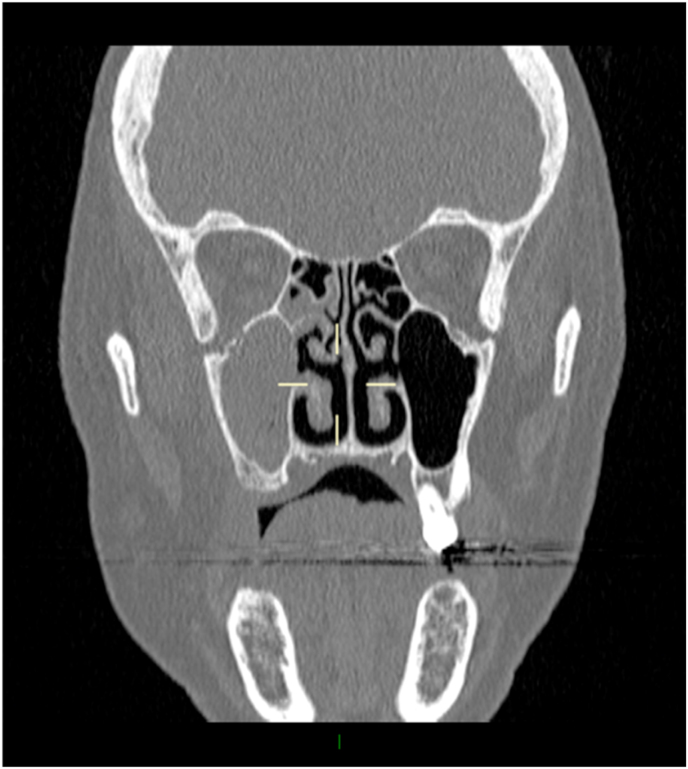
Evidence of right maxillary sinusitis with extension through the orbital floor (CT Sinus; coronal view).

Magnetic resonance imaging (MRI) of her head and neck on day 0 showed fluid in the right maxillary antrum with distortion of the orbital floor and elevation of the inferior rectus, with CT scan confirming right maxillary sinusitis but also showing extension into the orbit through the orbital floor (Fig. 1). An inflamed inferior rectus was noted with right-sided proptosis and inflammatory changes tracking into the pterygopalatine fossa. The patient was commenced on intravenous (IV) ceftriaxone 2g twice a day and metronidazole 500mg three times a day in addition to topical chloramphenicol 0.5% drops twice a day for a presumed right maxillary sinusitis with orbital cellulitis, given for a total of 7 days.

**Fig. 2:**
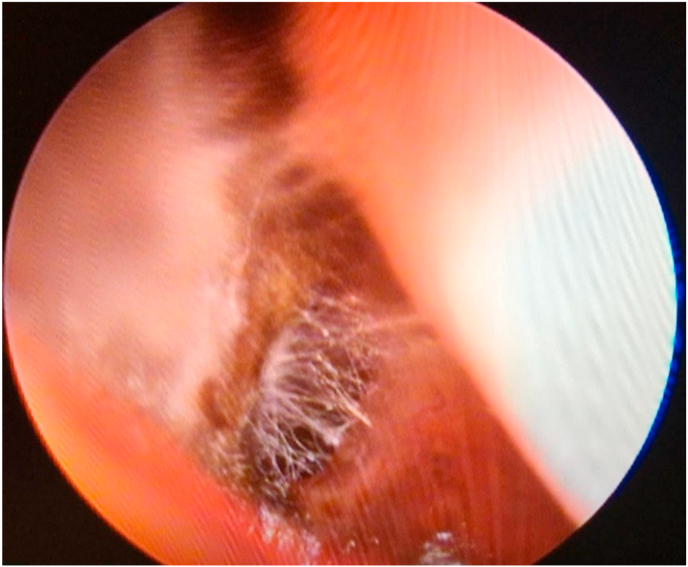
Appearance of mucosal fungal growth suggestive of an evolving fungal ball on flexible nasoendoscopy.

On day +2, the patient deteriorated with a decrease in conscious level, resulting in a GCS fluctuating between 13 to 15. She underwent maxillary sinus exploration and debridement, at which point endoscopic examination showed the presence of a fungus ball (Fig. 2; Video in Supplementary material section) with bony invasion of the skull base. Medial maxillectomy, anterior and posterior ethmoidectomy, right sphenopalatine artery ligation and medial orbital wall decompression were carried out. A diagnosis of invasive fungal sinusitis with skull-base osteomyelitis was made. After tissue samples were obtained, liposomal amphotericin B (L-AmB) was instilled into the maxillary sinus peri-operatively, and IV L-AmB) was then commenced at a dose of 5mg/kg daily. Euglycaemia was ensured through a variable-rate insulin infusion.

Supplementary video related to this article can be found at https://doi.org/10.1016/j.mmcr.2021.03.005

The following is/are the supplementary data related to this article:

Video 1 Video 1

**Fig. 3:**
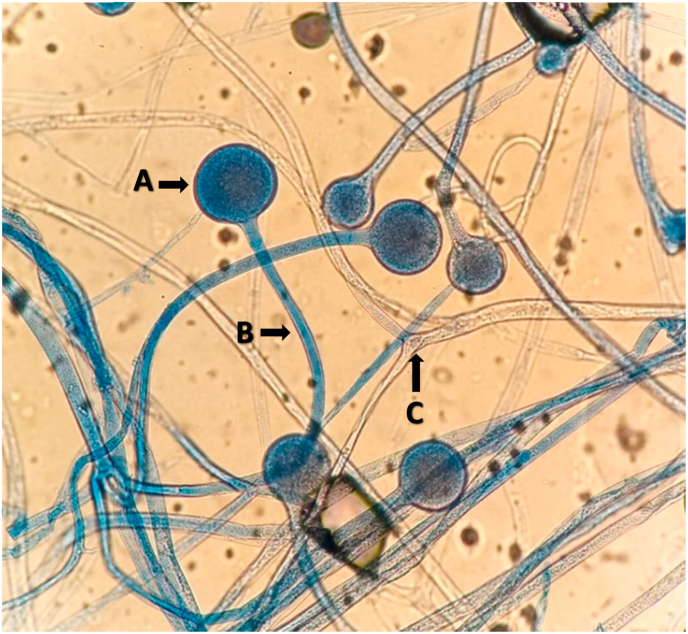
*Rhizopus arrhizus*, showing sporangium (A) on long sporangiophore (B) arising from broad, pauci-septate, ribbon-like hyphae branching at 90° (C) (**Lactophenol cotton blue stain, x100**).

Microscopy from tissue samples confirmed the diagnosis of mucormycosis, with the causative agent being identified as *Rhizopus arrhizus* (Fig. 3). This was identified by macroscopic and microscopic morphological examination with Lactofuchsin from cultures grown on Sabouraud's Dextrose agar supplemented with chloramphenicol (SABC, Oxoid). Species-level identification was confirmed by matrix-assisted laser desorption time of flight mass spectrometry (MALDI-ToF MS) using established methods [5] on the Bruker Biotyper Sirius, using Compass software (V4.1) with filamentous fungus database (V3). A Biotyper score of 2.17 was achieved, validating the identification to species level.

The following minimum inhibitory concentrations were established in the National Mycology Reference Laboratory using Clinical and Laboratory Standards Institute broth microdilution methodology: amphotericin B 0.06mg/L, posaconazole 0.25mg/L, isavuconazole 1mg/L, voriconazole 8mg/L, itraconazole 0.5mg/L.

The patient deteriorated further with decreased visual acuity of her right eye on day +10, with repeat flexible nasoendoscopy showing necrotic tissue and MRI revealing persistent intraorbital oedema involving both intra- and extra-conal spaces, with perineural enhancement of the right optic nerve extending from intra-orbital to intracranial part of the nerve, in keeping with the clinical suspicion of cerebral involvement. In view of this deterioration, a trial of dual-therapy involving the addition of IV posaconazole 300mg once daily was undertaken between days 8–13. This was subsequently stopped on day 5 of therapy in view of deranged liver function tests (Bilirubin: 30μmol/L; Alanine aminotransferase: 89 U/L; Aspartate aminotransferase: 224 U/L; Alkaline phosphatase: 1435 U/L; Albumin 16g/L) attributed to an acute drug-induced liver injury, with reversion to L-AmB monotherapy.

Further deterioration was noted on day +34, with repeat imaging showing intra- and extra-conal right orbital abscess formation, as well as new abscess formation in the right masticator space, associated with erosion of the right lateral maxillary wall. Given the extent of infection and lack of any alternative surgical options to achieve source control, exenteration of the right orbit was performed on day +36**.** Multiple abscesses were observed, necessitating further debridement of the upper lip, nasopharynx and oropharynx. Tissue samples from theatre yielded *Pseudomonas aeruginosa*, which was treated effectively with IV piperacillin/tazobactam 4.5g three times daily for 6 weeks. No fungal elements were observed on microscopy, with no further moulds being isolated from these samples despite prolonged incubation.

**Fig. 4:**
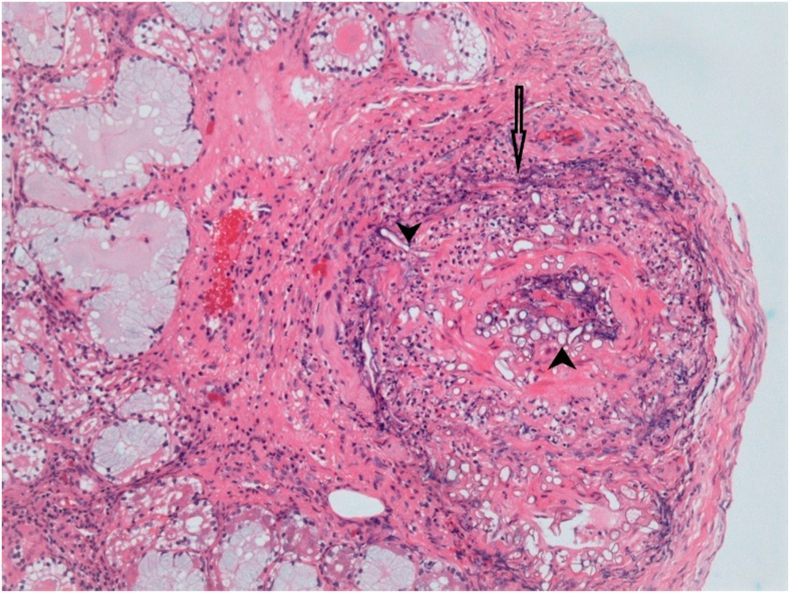
Histopathology of right lower eyelid/cheek subcutaneous tissue lined by markedly inflamed granulation tissue. Artery wall infiltrated by neutrophil polymorphs is denoted by the single arrow - diagnostic of vasculitis. The arrow heads indicate fungal hyphae present in the vessel lumen and infiltrating the vessel wall. **(Hematoxylin & eosin stain, x100)**.

Histological examination of intraoperative tissue samples revealed an inflammatory infiltrate consisting of a mixture of lymphocytes, histiocytes, neutrophils and multinucleated giant cells, with multiple foci of caseating granulomata seen in association with fungal hyphae, with focal intraneural and vascular invasion by fungal hyphae (Fig. 4). Acute osteomyelitis secondary to invasive mucormycosis was also confirmed histologically through the finding of foci of fungal hyphae and acute inflammatory infiltrate with bony necrosis in a tissue sample from the floor of orbit.

Satisfactory appearances were noted on subsequent examination under anaesthesia on day +38 & +42, with further local application of L-AmB onto wound packing. Her ongoing medical management was complicated by a gradual deterioration in her renal function secondary to L-AmB treatment. L-AmB was therefore stopped and isavuconazole 200mg once daily was introduced on day +37. Subsequent clinical deterioration led dual antifungal therapy being restarted, with further surgical debridement performed on day +54 and day +58. Tissue samples in these instances revealed glycopeptide-resistant *Enterococcus faecium* (GRE)*,* treated with IV daptomycin at 10mg/kg once daily for a total of 6 weeks.

The patient's clinical condition slowly improved, albeit with ongoing renal impairment. L-AmB was stopped and isavuconazole was converted to enteral formulation on day +85. Her progress was complicated by an intravascular catheter-related bloodstream infection secondary to *Klebsiella pneumoniae* on day +90, which was managed effectively with 7 days of IV meropenem 1g three times daily. Her insulin regimen was converted to a twice daily subcutaneous insulin regime on day +105. Repeat MRI sinus on day +121 showed no significant disease progression, and the patient was discharged on day +138 on a prolonged enteral course of isavuconazole 200mg once daily. On last outpatient review, the patient was clinically well with no evidence of disease recurrence. Facial reconstruction by the Maxillo-Facial team was subsequently undertaken with a satisfactory outcome.

## 3 Discussion

This case is noteworthy in highlighting a number of challenges encountered in the management of mucormycosis: initial diagnostic uncertainty, the need for aggressive surgical intervention, the importance of risk-factor modification and aggressive treatment of bacterial co-infections, as well as difficulties around drug toxicity. It also illustrates the utility of salvage treatment with the recently-introduced triazole isavuconazole, which was significantly better tolerated than L-Amb and posaconazole.

Early diagnosis and initiation of effective antifungal therapy has been shown to improve outcomes in mucormycosis, with delayed amphotericin B therapy (≥6 days after diagnosis) resulting in an almost twofold increase in mortality at 12 weeks in this retrospective study [6]. In this case, the initial findings of pus and oedema but no tissue necrosis or eschars on flexible nasoendoscopy led to a working diagnosis of bacterial sinusitis with skull base osteomyelitis, resulting in a 48-h delay to start effective therapy despite the presence of classical risk factors for mucormycosis. This highlights the importance of hypervigilance in securing early diagnosis and treatment, as well as the dangers of excluding this diagnosis based on absence of initially-negative endoscopic appearances.

Surgical intervention with removal of necrotic tissue has likewise been associated with improved survival in this anecdotal clinical review of rhinocerebral infection [7]. As in this case, this may come at a price of surgical intervention that is often disfiguring, requiring removal of the palate, nasal cartilage, and the orbit, with subsequent reconstruction being required. Endoscopic debridement with limited tissue removal can be accomplished in select cases, however in the case presented ongoing deterioration despite appropriate antimicrobial therapy necessitated radical open debridement.

Diabetes mellitus is the most common underlying condition in those with mucormycosis, occurring in 36–40% of cases and is associated with increased mortality [2,8] ROCM is observed significantly more in diabetic patients, with 60–88% of those developing ROCM having diabetes [8,9]. Microvascular disease in diabetes is thought to be associated with a fragile sinus architecture, with increased susceptibility to local spread [2]. Hyperglycaemia has also been shown to inhibit both innate immunity, through polymorphonuclear neutrophil and macrophage dysfunction failing to suppress spore germination as well as adaptive immunity [10,11]. This is also borne out by the observation of bacterial co-infection with *P. aeruginosa* and GRE.

Hyperglycaemic control is strongly recommended in the 2013 ESCMID and ECMM joint clinical guidelines for the diagnosis and management of mucormycosis and indeed tighter glycaemic control was accompanied by an improved clinical response in our patient [3].

Drug toxicity was the main consideration behind decisions regarding optimal anti-fungal therapy. Despite decreased rates of nephrotoxicity with L-AmB compared to amphotericin B deoxycholate, this remains a considerable limitation, leading to worsening renal function in up to 29% of patients in one study, with 8% classed as moderate-severe dysfunction [12]. A dose-dependent relationship exists, with 10mg/kg regimens having significantly higher rates of nephrotoxicity when compared to 3mg/kg, despite no significant improvement in outcomes between the groups [13].

Both posaconazole and isavuconazole are broad-spectrum azoles with activity against the agents of mucormycosis, and the availability of an oral formulation makes them an attractive oral step-down therapy for patients who have responded to treatment. In our case, combination therapy with L-AmB and posaconazole was attempted, although there are currently no studies to support this [14]. Posaconazole was discontinued after 6 days in view of drug-induced liver injury. This was a marked transient elevation in serum aminotransferase and bilirubin levels rather than clinically-apparent hepatotoxicity, with the latter being very rare. In view of its discontinuation within less than 7 days of treatment, no therapeutic drug monitoring was performed for this agent.

Isavuconazole was subsequently used, initially as an IV agent in combination with L-AmB on day +37, and subsequently as a step-down option on day +85. It was tolerated well, in keeping with its favourable pharmacokinetic and safety profile compared to other antifungals [15]. Therapeutic drug monitoring was not performed as concentration distributions from real-world use and clinical trials do not suggest it to be routinely required [16,17], and initial treatment failure while on isavuconazole was attributed to further debridement being required as well as GRE co-infection. Isavuconazole has been shown to be efficacious for both primary and salvage therapies, with overall end-of-treatment response rates comparable to L-AmB, and no difference in crude or all-cause mortality [18].

In summary, we present a case of successful treatment of ROCM with a combination of aggressive surgical debridement and L-AmB followed by isavuconazole as salvage anti-fungal therapy. This case adds to the growing evidence supporting the use of isavuconazole in invasive mucormycosis.

## Funding source

There are none.

## Consent

Written informed consent was obtained from the patient or legal guardian(s) for publication of this case report and accompanying images. A copy of the written consent is available for review by the Editor-in-Chief of this journal on request.

## Declaration of competing interest

There are none.
